# Surgical amputation for patients with diabetic foot ulcers: A Chinese expert panel consensus treatment guide

**DOI:** 10.3389/fsurg.2022.1003339

**Published:** 2022-11-08

**Authors:** Xuan Liao, Sheng-Hong Li, Mariya Mohamad El Akkawi, Xiao-bing Fu, Hong-wei Liu, Yue-sheng Huang

**Affiliations:** ^1^ Department of Plastic Surgery of the First Affiliated Hospital of Jinan University, Institute of New Technology of Plastic Surgery of Jinan University, Key Laboratory of Regenerative Medicine of Ministry of Education, Guangzhou, China; ^2^ Wound Healing and Cell Biology Laboratory, Institute for Basic Research, Trauma Center of Postgraduate Medical College, General Hospital of PLA, Beijing, China; ^3^ Department of Wound Repair; Institute of Wound Repair and Regeneration Medicine, Southern University of Science and Technology Hospital, Southern University of Science and Technology School of Medicine, Shenzhen, China

**Keywords:** diabetic foot management, diabetic foot ulcer, amputation, wound healing, expert consensus

## Abstract

**Background:**

Diabetic foot disease is a serious complication of diabetes mellitus. Patients with diabetes mellitus have a 25% lifetime risk for developing a foot ulcer, and between 14% and 24% of patients require a major or minor lower limb amputation due to severe gangrene. However, decisions concerning whether to amputate or whether to perform a major or minor lower limb amputation, and how best to determine the amputation plane remain unclear.

**Methods:**

To consolidate the current literature with expert opinion to make recommendations that will guide surgical amputation for patients with diabetic foot ulcers. A total of 23 experts experienced in surgical treatment of patients with diabetic foot ulcers formed an expert consensus panel, and presented the relevant evidence, discussed clinical experiences, and derived consensus statements on surgical amputation for patients with diabetic foot ulcers. Each statement was discussed and revised until a unanimous consensus was achieved.

**Results:**

A total of 16 recommendations for surgical amputation for patients with diabetic foot ulcers were formulated. The experts believe that determination of the amputation plane should be comprehensively evaluated according to a patient's general health status, the degree of injury, and the severity of lower limb vasculopathy. The Wagner grading system and the severity of diabetic lower extremity artery disease are important criteria when determining the degree of amputation. The severity of both diabetic foot infection and systemic underlying diseases are important factors when considering appropriate treatment. Moreover, consideration should also be given to a patient's socioeconomic status. Given the complexities of treating the diabetic foot, relevant issues in which consensus could not be reached will be discussed and revised in future.

**Conclusion:**

This expert consensus could be used to guide doctors in clinical practice, and help patients with diabetic foot ulcers gain access to appropriate amputation treatment.

## Introduction

Diabetes mellitus (DM)-related morbidity rates are increasing globally (1), with a further predicted increase from 5.1% to 7.7% by 2030 (2). China has the highest DM-related morbidity rate in the world. The 2020 Guidelines for the Prevention and Treatment of Type 2 Diabetes in China reported that the incidence of DM in China has continued to increase (9.1%, 10.4%, and 11.2% in 2010, 2013, and 2017, respectively) and that DM is the second most common chronic disease after hypertension (3). Among the affected population in China, factors such as low awareness, poor prevention strategies, poor DM control, DM-related complications characterized by a high incidence, a long disease duration, and the presence of other serious complications significantly impact patient quality of life as well as public health resources and expenditure. “The diabetic foot” describes a foot with infection, active wound formation, and/or deep tissue destruction due to DM-related neuropathy and/or peripheral artery disease, which is the most common major end-point of DM-related complications (4). The lifetime risk for developing a DM-related foot ulcer has been reported to be as high as 25%. Peripheral neuropathy, vasculopathy, and trauma are the main contributing factors in DM-related foot disease. Healing DM-related foot ulcers is challenging and between 14% and 24% of patients with foot ulcers have been reported to undergo minor amputation (5). Although expert consensus or guidelines on the treatment of diabetic foot disease and basic principles of minor or major lower limb amputation have been published, many challenges remain concerning decisions on amputation or salvage, minor or major limb amputation, and evaluation and determination of the amputation plane for patients with diabetic foot disease. Considering that the five-year survival rate of patients with major amputations is reported to be 30%, while the three-year survival rate of patients with minor amputations or non-amputations is 93% (6), it is necessary for both patients and surgeons to carefully evaluate decisions concerning amputations for patients with diabetic foot disease. Therefore, a summary of issues concerning DM-related lower limb amputations and a unified understanding are required. In this consensus treatment guide, we analyzed and summarized studies concerning treatment of the diabetic foot, and we discussed and standardized factors related to amputation. Given the complex etiology of the diabetic foot, treatment should be made in consideration of a patient's past history and overall health status, including a patient's socioeconomic status. For issues where no consensus was reached, further revisions will be undertaken based on evidence-based, multi-centered, large-sample clinical trials, in addition to consultation with experts working in relevant fields.

## Methods

The following databases were searched: PubMed, Cochrane Database, The National Institute for Health and Care Excellence (www.nice.org.uk), Wanfang, and China National Knowledge Infrastructure (CNKI). Randomized controlled studies, cohort studies, case-control studies, case-historical controls, cross-sectional studies, and meta-analyses related to the clinical treatment of diabetic foot disease were retrieved using the following keywords: “diabetic foot”, “foot ulcer”, “amputation”, “wound healing”, and “consensus”. Any differences between the search and selection of literature were determined by a third external reviewer.

According to the principles of the Participant, Intervention, Comparisons, and Outcomes resource (PICO), a literature search on the clinical data of diabetic foot amputation was conducted in the corresponding databases (Population: patients with diabetic foot disease; Intervention: evaluation and clinical treatment of the diabetic foot; Control: DM-related lower limb amputation; Results: level of evidence and degree of recommendation level). Experimental data included in each study were screened, and the evidence quality was rated. Experts who participated in the development of the consensus treatment guide made a comprehensive evaluation of each outcome index, and finally made corresponding judgments and recommendations for DM-related amputations.

In terms of evidence quality rating, the GRADE (Grades of Recommendations Assessment, Development, and Evaluation) system was used to divide the evidence quality into four grades: high, medium, low, and very low. Randomized controlled trials (RCTs) without obvious design defects were graded as providing the highest level of evidence, whereas observational trials were graded as providing the lowest level of evidence. If factors were present in RCTs that might decrease the quality of the evidence, then these RCTs were downgraded accordingly. If factors were present in observational trials that might increase the quality of the evidence, then these trials were upgraded. A unified GRADE quality assessment was undertaken for all original studies. Following preliminary grading according to the original study design type, upgrade or downgrade factors were fully considered. Ultimately, the quality of evidence for each original study fell into one of four categories ranging from high to very low (7).

The development of this consensus treatment guide was based on current evidence and clinical practice data, combined with the experience and opinions of wound repair experts engaged in diabetic foot treatment and management, with the aim of providing an academic basis and guiding opinions for clinical practice in treating diabetic foot disease.

## Results

1. **DM-related surgical amputation, including open and closed lower limb amputations, based on wound status.**

In a 2019 (No. 865) notice issued by the Chinese National Health Committee, diabetic foot treatment and DM-related foot amputations are required to be undertaken by wound repair specialists.

Lower limb amputations are divided into open and closed amputations according to the timing and conditions of the operation. Selection of an open or closed amputation should be undertaken in relation to the presence of local infection, tissue ischemia, and systemic conditions.

DM-related lower limb amputations are divided into major and minor amputations according to the plane of amputation. Major amputation refers to an amputation above the ankle when alleviation of the severe disease state through vascular remodeling, drug control, or minor amputation is not possible. Minor amputation refers to an open or closed local amputation with limited tissue excision, normally at the level of the ankle or below, employing partial vascular reconstruction or limb correction while removing infected and necrotic tissue.

DM-related lower limb amputation greatly affects a patient's lifestyle and prognosis, particularly major amputations, and should be carefully evaluated.

2. **Given the complex etiology of the diabetic foot, selecting the amputation type and determination of the amputation plane should be comprehensively evaluated according to the degree of injury, the severity of lower limb vasculopathy, the patient's overall systemic condition, and other indicators.**

Minor DM-related amputations include digital amputation, metatarsal resection, and partial foot amputation. Digital amputations may be performed in cases involving deep toe ulcers or bone destruction. Metatarsal resection may be used to treat DM-related ischemic necrosis of a digit combined with osteomyelitis of an adjacent metatarsal bone. When three or four toes require amputation, a transmetatarsal amputation may be performed directly to ensure better static stability of the affected foot. When gangrene of the foot extends proximally and there is a lack of suitable soft tissue to cover the metatarsal shaft, a mid-foot amputation may be considered. A Syme amputation may be considered when extensive foot gangrene or infection cannot be resolved through mid-foot amputation. A Pirogoff amputation may be considered for a patient whose forefoot cannot be reconstructed but whose posterior foot remains relatively intact.

Major amputations include below-knee amputations, above-knee amputations, and hip amputations. A major amputation may be considered following a minor amputation if the wound is less likely to heal, if gangrene or infection has spread to the mid-foot region, or if the patient has lost mobility. In patients with distal arterial occlusive disease and foot gangrene, a below-knee amputation is an option. An above-knee amputation may be considered for older patients with complete occlusion of the popliteal artery where reconstruction of the inferior popliteal artery is not possible, for patients with a flexion contracture deformity of the knee, and for patients deemed unsuitable candidates for multiple surgical procedures.

**Summary:** This consensus treatment guide was mainly formulated around clinical operability; therefore, there are some limitations. We would anticipate that experts engaged in wound repair could provide further opinions and suggestions.

3. **The Wagner grade for diabetic foot disease is currently the most widely used rating system for the evaluation of the diabetic foot, and is an important basis for determining DM-related amputation treatment.**

The higher the Wagner grade was, the greater was the possibility of amputation, and the lower was the cure and improvement rates (8, 9). A lower limb amputation should be selected for patients with Wagner Grade 5 lesions. Amputations may be selected according to the general condition and the site of gangrene for patients with Wagner Grade 4 lesions. If a patient's general condition is poor and gangrene is located in the toe, then amputation should be selected. For patients with Wagner Grade ≤3 diabetic foot lesions, limb salvage therapy should be attempted whenever possible.

4. **The severity of DM-related lower extremity artery disease is an important factor when deciding to amputate.**

DM-related lower extremity vasculopathy is an independent risk factor for diabetic foot disease, and can lead either to delayed healing of wounds in the lower extremity or directly to lower extremity ischemia and necrosis. The severity of diabetic foot lower extremity vasculopathy is also an important factor when deciding whether to amputate, particularly when this complication is combined with a history of stroke, coronary heart disease, or other cardio- or cerebrovascular diseases.

**Summary:** The prevalence of lower extremity vasculopathy in patients with DM increases with age and DM duration, and is an independent risk factor for diabetic foot disease that can lead either to delayed healing of lower extremity ulcers or directly to lower extremity ischemia and necrosis (10). In China, the incidence of lower extremity vasculopathy in hospitalized patients aged ≥50 years with DM has been reported to be as high as 19.47% (11). Other studies have shown that the prognosis for patients with diabetic foot disease complicated with lower extremity vasculopathy is poorer than that for many common cancers, and the five-year mortality has been shown to be as high as 50% (12). Amputation may be selected when a diabetic foot and lower limb vasculopathy is associated with such cardiovascular risk to reduce the risk for cardiovascular death (13, 14).

5. **The severity of diabetic foot infection is an important reference factor in the choice for DM-related minor or major amputations.**

Diabetic foot infection is based on a clinical diagnosis of local and systemic inflammatory responses. Use of the International Working Group on Diabetic Foot (IWGDF)/Infectious Diseases Society of America (IDSA) grading standard to determine the severity of diabetic foot infection is suggested. The severity of diabetic foot infection is an important reference factor in the choice of whether to amputate for patients with diabetic foot infection. The higher the grade of diabetic foot infection was, the greater was the possibility of minor or major lower limb amputation and the worse was the prognosis (15). Amputation should be considered for patients with diabetic foot disease and severe infection (Wagner Grade ≥3).

**Summary:** A retrospective study of IWGDF/IDSA grading criteria for diabetic foot infection showed that when the infection grade was higher, the duration of antibiotic use was longer, the frequency of operations was higher, the number of operations was greater, the percentage of amputations was higher as was the incidence of reinfection, and the length of the hospital stay was longer. That study also found that the IWGDF/IDSA grading standard better reflected the prognosis for patients with diabetic foot disease.

6. **In patients with diabetic foot disease, systemic disease severity status is also an important consideration when deciding whether to amputate.**

Patients with diabetic foot disease require a comprehensive assessment of any other underlying systemic complications, particularly concerning cardiovascular, cerebrovascular, and renal disease, and assessment of other important organ functions. For patients with end-stage renal failure and long-term hemodialysis, amputations should be considered for patients with Wagner Grade ≥3 wounds.

**Summary:** A comprehensive patient assessment aids in diagnosing and treating a patient with diabetic foot disease and in determining an accurate prognosis, as well as reducing treatment-related risks and complications. A history of DM is associated with a 2–4 times increased independent risk for cardio- and cerebrovascular disease. The incidence of heart failure in patients with diabetic foot ulcers is reported to be 39%, and the incidence is related to the severity of foot lesions (16). Among the complications of DM, chronic renal failure is the most serious risk factor for DM-related lower limb amputation (17), with a reported amputation healing rate of 50%–60%, together with an increased risk for postoperative bleeding and hematoma formation due to hemodialysis (18).

7. **Lower extremity revascularization is an effective method for treating lower extremity vascular occlusion.**

When diabetic foot infection is complicated with peripheral artery disease, lower limb revascularization (bypass grafting and endovascular therapy) should be performed to restore blood circulation in ischemic limbs and direct blood flow to at least one pedal artery, which is necessary for limb preservation. However, lower extremity revascularization, particularly endovascular therapy, is associated with a high risk for re-occlusion in patients with DM-related pedal artery occlusion. Therefore, the potential benefits of lower extremity revascularization should be carefully evaluated to determine whether minor or major lower limb amputation is indicated when re-occlusion occurs.

**Summary:** Lower extremity revascularization has been shown to achieve a limb salvage rate of 80%–85% and an ulcer healing rate of >60% within 12 months postoperatively (19). The results of one study involving 101 recanalization procedures indicated that there was no difference between single and multiple recanalization regarding ulcer healing rates at 12 months and limb preservation rates at 24 months (20). This suggested that for patients with DM and lower extremity ischemic ulcers, better results can be expected when an open vessel is ensured and blood flow is restored to the ischemic region of the foot.

8. **Given the complexity of treatment for diabetic foot disease, wound repair specialists should jointly consult with physicians of related disciplines to develop an optimal plan to reduce the amputation plane and amputation and mortality rates for patients with DM, and improve wound healing rates post-amputation.**

**Summary:** A multidisciplinary team approach has been shown to contribute to reduced amputation rates and treatment costs, particularly one that involves surgeons, with a reported reduction in major amputations of 82% (21–27).

9. **Good blood glucose control could promote diabetic foot ulcer healing, reduce the risk for wound infection and amputation, and reduce the risk for wound infection post-amputation, facilitating stump wound healing.**

**Summary:** Patients with higher hemoglobin A1c (HbA1c) levels have been found to have significantly longer ulcer healing times (28, 29). Blood glucose levels are closely associated with prognosis for patients with diabetic foot disease. A study analyzing nine RCTs regarding the relationship between different blood glucose levels and diabetic foot ulcer healing showed that, among 10,897 patients with type 2 DM, patients with enhanced blood glucose control (HbA1c range, 6.0%–7.5%) had a lower amputation rate (relative risk [RR], 0.65; 95% confidence interval (CI) 0.45–0.94; *P* = 0 and a slowly decreasing sensory threshold (mean difference, 8.27; 95% CI 9.75–6.79) (30).

10. **Percutaneous oxygen partial pressure measurement, which can also be combined with vascular imaging, is recommended to determine the plane of amputation in diabetic foot disease.**

An amputation stump will not heal when the tissue percutaneous oxygen partial pressure is <20 mmHg. However, an amputation stump can heal when this is >40 mmHg. Healing potential is possible between these values; however, interventions to increase blood flow are required. For minor amputations, when a preoperative blood perfusion assessment of the affected limb indicates an acrotarsium percutaneous oxygen partial pressure of ≥30 mmHg and a segmental perfusion pressure of ≥70 mmHg, the wound healing rate is increased.

**Summary:** On the basis of existing reports and in consideration of factors such as personnel, equipment, time, and cost availability, together with confirmation or otherwise of the presence of invasive infection into deep tissue and bone, percutaneous partial oxygen pressure is currently the most recommended method for determining the amputation level.

11. **When phalanges are involved, affected toe tissue can be surgically removed, sufficient tissue should be retained at the stump for a skin flap, wound closure should be performed in accordance with the tension-free principle, and, if necessary, bone tissue may be sacrificed.**

**Summary:** Deep toe ulcers and accompanying phalangeal osseous destruction require amputation involving the articular surface or part of the toe. When conservative treatment fails, toe ulcers can lead to bone infection and destruction, which requires amputation of part or all of the toe. Not all toe amputations are undertaken at the metatarsophalangeal joint level, because it may be possible to eliminate the infected site completely while salvaging sufficient toe tissue to serve as a shock absorber between the adjacent toes. Primary closure of the surgical wound can be undertaken in partial toe amputation, and the stump should retain sufficient flap pedicle to cover the bone; therefore, it is occasionally necessary to make an incision at the edge of the necrotic tissue or at the ulcer edge. There is a balance to be struck between preserving bone and preserving sufficient skin and soft tissue to ensure that a surgical wound can be closed in accordance with the tension-free principle, and part of the bone can be sacrificed if necessary (31, 32).

12. **For closed amputations, suturing the amputation stump site may affect blood flow to the region; therefore, full layer suturing is preferred. To reduce the influence of sutures on a wound's blood supply, the needle distance of the suture should be increased as far as possible while ensuring wound alignment, and negative pressure suction post-amputation and suturing are suggested.**

**Summary:** For ideal wound repair, particularly for the diabetic foot postoperatively, adjacent autogenous tissue should be used to repair the wound without tension after first-stage debridement and wound sutures have been placed, and the repaired tissue should be able to withstand continuous pressure and shear force in all directions during standing and walking. The sequence of repair techniques from simple to complex are as follows: closure by primary intention, closure by secondary intention, negative pressure wound therapy (NPWT), skin graft, dermal matrix graft, local flap transplantation, distal flap transplantation, tissue expansion, local fascial or myofascial flap, island flap, and free tissue transplantation (33). The selection of diabetic foot wound repair techniques should be undertaken in a step-by-step manner in accordance with the above order, with a preference for simple solutions to complex ones. An analysis of four low-to-moderate-quality RCTs showed that NPWT promoted granulation proliferation and wound healing. One study reported that the wound healing rate increased by 20% (odds ratio, 2.0%; 95% CI −1.0–4.0) and that the amputation rate decreased by 7.9% (34, 35).

13. **Compared with other anesthesia methods, a peripheral nerve block is the first-choice anesthetic method for DM-related amputation, because this method has been shown to better stabilize hemodynamics and control postoperative pain.**

**Summary:** In the surgical treatment of the diabetic foot, a peripheral nerve block is the first choice of anesthesia, because it has been shown to better stabilize hemodynamics (36) and control postoperative pain (37–40).

14. **Antibiotic therapy should be administered post-amputation.**

The use of antibiotics should be based on the severity of clinical infection, bacterial culture results, drug sensitivity test results, and a comprehensive assessment of a patient's liver and kidney functions.

**Summary:** Antibacterial treatment should be administered on confirmation of a clinically infected diabetic foot wound. Prior to the confirmation of bacterial culture and drug sensitivity test results, empirical medication may be administered according to the clinical manifestations of infection and a comprehensive evaluation of hematological indexes and liver and kidney functions (41). After obtaining the results of bacterial culture and drug sensitivity tests, antibiotic therapy needs to be adjusted accordingly. For patients with DM-related lower limb osteomyelitis, antibiotic therapy for 2–5 days is recommended for those who have undergone surgical debridement without postoperative residual tissue infection. Patients with residual soft tissue infection should be treated with antibiotic therapy for 2–4 weeks, while those with residual bone infection should be treated with antibiotic therapy for 4–6 weeks (42–45).

15. **The healing of diabetic foot wounds should be evaluated during different periods when treating patients with active diabetic foot disease. If a wound area has reduced by 10%–15% within one week or by >50% within four weeks, limb salvage treatment can be continued; otherwise, minor or major lower limb amputation may be required.**

**Summary:** Most diabetic foot ulcers display a dynamic change trend. One study showed that if the area of an ulcer decreased by 10%–15% within one week or by >50% within four weeks, the possibility of reinfection and amputation was significantly reduced, indicating that the percentage of ulcer area reduction per unit time has an early predictive value for the curative effect (46–49).

16. **During the treatment of diabetic limb salvage, full consideration should be given to the benefits of limb salvage or recanalization.**

In cases of complications due to cardiovascular disease, amputation above the occlusion level may be considered to reduce the amputation plane (except in the absence of a femoral or popliteal artery pulse). The plane of vascular occlusion should be determined by using color duplex ultrasound, digital subtraction angiography, magnetic resonance angiography, and computed tomography angiography (CTA).

**Summary:** The amputation rate has been reported to increase in patients with severe limb ischemia who have not received timely vascular revascularization (a delay >2 weeks) (50). However, patients with ischemic diabetic foot disease that is complicated with infection need to undergo urgent assessment and treatment of their systemic status and of the wound surface, which are challenging because the risk for amputation or mortality is high (a perioperative mortality rate of 5%). One study reported a one-year limb salvage rate post-vascular reconstruction of approximately 70%; however, the one-year mortality rate was approximately 40% (51). Several observational studies have shown that in patients with severe ischemic diabetic foot ulcers without revascularization, the ulcer healing rate (with or without minor amputation) is almost 50%. Therefore, when the risk for revascularization is high and the risk-benefit ratio is unclear, clinical decision-making should include careful consideration of the treatment benefits, detailed preoperative discussion of the treatment plan, and patient and family communication regarding the surgical plan, including cardiovascular examination, electrocardiogram, echocardiography, and cardiac CTA or coronary angiography findings, if needed. Revascularization should be avoided and amputation should be undertaken for patients with an adverse risk-benefit ratio in terms of the surgical success rate.

## Conclusion

DM-related minor or major amputations of the lower limb are known to significantly affect patient prognosis and quality of life; therefore, a multidisciplinary approach should be recommended to form an optimal treatment, in addition to careful determination of the amputation type and plane. Diabetic foot amputation should be comprehensively evaluated according to a patient's general health status, the degree of injury, the severity of lower limb vasculopathy, and other relevant indicators. The Wagner grading system of diabetic foot and the severity of diabetic lower extremity artery disease are important bases for determining amputation (toe) of diabetic foot. The severity of both diabetic foot infection and systemic underlying diseases are important factors when considering appropriate treatment. Moreover, consideration should also be given to a patient's socioeconomic status. Given the complexities of treating the diabetic foot, relevant issues in which consensus could not be reached will be discussed and revised in future through multi-center, large-sample clinical observation studies and expert opinion.

## Data Availability

The original contributions presented in the study are included in the article/Supplementary Material, further inquiries can be directed to the corresponding author/s.
